# The impact of climate change on hospice and palliative medicine: A scoping and narrative review

**DOI:** 10.1016/j.joclim.2024.100323

**Published:** 2024-05-25

**Authors:** David Harris, Bhargavi Chekuri, Aldebra Schroll, Nisha Shah, Laadi Swende, Collins Uzuegbu, Pamela Young

**Affiliations:** a Department of Palliative and Supportive Care, Taussig Cancer Institute, Cleveland Clinic, CA53, 9500 Euclid Ave, Cleveland, OH 44195, United States; b University of Colorado School of Medicine, Aurora, Colorado, United States; c Butte Home Health and Hospice, Chico, California, United States; d Section of Palliative Care and Medical Ethics, Division of General Internal Medicine, Department of Medicine, University of Pittsburgh Medical Center, Pittsburgh, Pennsylvania, United States; e National Health Insurance Authority, Federal Medical Centre, Makurdi, Nigeria

## Abstract

**Introduction:**

Climate change's severe impact on human health is becoming increasingly evident, particularly for vulnerable populations with serious illnesses. Climate-related extreme weather events are expected to increase demand for hospice and palliative care due to rising respiratory illnesses, heat-related issues, waterborne diseases, and aggravated chronic conditions. Our scoping review aimed to investigate the existing literature on climate change's impact on hospice and palliative medicine (HPM).

**Methods:**

We conducted a comprehensive literature search across various databases (e.g., Medline, EMbase, Web of Science, and Cochrane) using predefined climate change and HPM terms, resulting in 382 records. Following predetermined inclusion and exclusion criteria, 44 articles were selected for full-text review, and 20 were included for final analysis. In light of the limited literature on climate change's impact on HPM, we also sought narratives from HPM practitioners from across the world on their experiences in a changing climate.

**Results:**

Six major themes emerged: 1) The impact of climate change on HPM in low-income countries; 2) Descriptive pieces on climate change, climate disasters, and HPM; 3) Morbidity and mortality after climate disasters in the seriously ill population; 4) Discussion of euthanasia during climate disasters; 5) Recommendations and frameworks for disaster response in the field of HPM; 6) Carbon footprint of hospices. Additionally, narratives from HPM practitioners highlighted the disruptive effects of climate disasters on seriously ill patients and their caregivers as disasters caused care interruptions, reduced access to crucial health infrastructure, exacerbations of illness, accelerated disease progression, and increased morbidity and mortality.

**Conclusion:**

Existing research on climate change's impact on HPM is primarily anecdotal and descriptive, with a focus on climate-related disasters. Narratives from HPM practitioners worldwide underscore the disproportionate impact of climate disasters on seriously ill patients. Further research is necessary to comprehensively understand climate's intricate effects on HPM and to assess adaptable, mitigative, and resilient solutions against its adverse impacts.

## Highlights

• Most articles are descriptive or offered recommendations for disaster preparedness.
• Common barriers to patient care include physical access to patients, lack of power for medical equipment, and provider distress.
• Identified future directions for research on the intersection of climate change and the field of hospice and palliative medicine.

## 1 Introduction

In 2023 alone, mankind bore witness to extreme heatwaves in North America, Europe and Asia, [1] unrelenting Canadian wildfires burning over 13 million hectares of land, [2] drought in Africa and Central America, and flooding events [3] around the world – all fueled by anthropogenic climate change [4]. As communities and governments contend with these challenges, the devasting impact of climate change on human health has never been more apparent. Scientists expect that without concerted efforts to rapidly reduce greenhouse gas emissions, Earth's temperature will rise by more than 1.5 °C leading to continued environmental degradation and even more frequent and intense climate-related disasters [4]. These profound environmental changes are harmful to human health through numerous direct and indirect pathways, and threaten many global public health improvements of the 20th and 21st century [5]. As with many other social and environmental determinants of health, climate change does not impact everyone equally. There are mounting data that undeniably show how the most vulnerable and marginalized populations of society are disproportionately affected by the climate crisis [4,5]. This includes those living with serious and life-limiting illnesses.

Palliative care is a field of medicine that provides specialized care for those living with serious, complex, and life-limiting illnesses. It takes an interdisciplinary approach to optimize the quality of life of patients and to ease their suffering. Palliative medicine aims to care holistically for patients with life-limiting illness and their loved ones by addressing their physical, psychological, social, and/or spiritual well-being. Hospice services are a continuum of palliative care and specifically provide specialized end-of-life care. It is estimated that close to 60 million people (including about 26 million people in their last year of life) need palliative care services [6]. The need for palliative care inevitably will grow in the setting of the climate-driven increases in serious respiratory illness; heat-related illness; water, food, and vector-borne diseases; and even exacerbated noncommunicable diseases.

To better understand the impact of climate change on seriously ill patients and clarify the need for further research and solutions, this review aims to investigate the existing literature of climate change's impact on hospice and palliative medicine (HPM). We also sought narratives from HPM practitioners across the world to share their experiences in a changing climate.

## 2 Methods

### 2.1 Search strategy

We conducted a search using MeSH terms for climate change, hospice and palliative care, employing truncation and adjacency for keyword searches to enhance the potential relevance of results. The search covered the period from inception of the databases through June 22, 2023. The selected databases for this search included Medline ALL® and Embase from Ovid, Web of Science Core Collection from Clarivate, and Cochrane Central Register of Controlled Trials via Wiley. Conference and meeting abstracts were excluded whenever possible through a publication filter, and the results were limited to English language publications. Of note, our scoping study was not registered with PROSPERO.

**Fig. 1:**
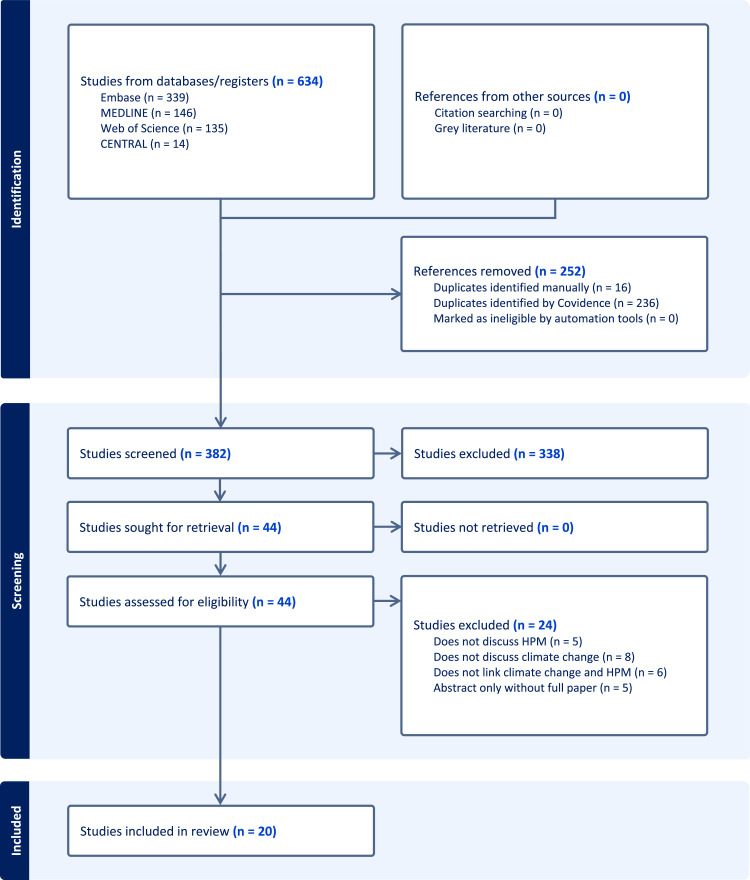
PRISMA flow diagram.

The identified articles were imported and stored using Covidence, a citation manager used for systematic reviews [7]. The search yielded a total of 634 results (Fig 1). Covidence removed 236 duplicates and another 16 duplicates were manually removed. Each title and abstract were reviewed by two members of the research team (DH, CU, NS, PY, LS, AS) and disagreements were arbitrated by a third member. Screening criteria required the title and abstract to discuss the impacts of climate change and discuss either hospice, palliative care, or end-of-life care. Articles that passed through the title and abstract screen underwent a full text review by two separate members of the team (DH, CU, NS, PY, LS, AS) and disagreements were again arbitrated by a third member. Full text inclusion criteria were similar to the title and abstract screening criteria. Articles were included if, in their full text, they discussed the impacts of climate change and hospice care, palliative medicine, or end of life care, and linked the two together. They were excluded if they were animal studies, abstracts without full papers, or articles where the full text was not available. There was no assessment of study quality as this was a scoping review.

### 2.2 Data extraction and synthesis

Data elements designated for extraction were chosen through group consensus. They were evaluated and revised iteratively during the data extraction process. Data from each article were extracted twice by separate members of the team. Items for extraction included title, authors, year published, type of paper, and the key points made in the article related to our research question. Because most papers chosen for extraction were perspective pieces, information around methodology was not captured for all pieces. Once data extraction was completed, the group engaged in discussion to achieve consensus on each aspect of the extraction. Relationships between articles were explored, and patterns across articles were identified. Through a group process, themes present across the included publications were identified and refined.

### 2.3 Narrative component

Because our scoping review was relatively limited in the number of articles identified, we supplemented the literature review by reaching out through formal and informal networks to HPM providers whose work was affected by climate change to request narratives of their experience, including two presented in this article.

## 3 Results

Our initial search strategy yielded 634 papers. After duplicates were removed, 382 papers remained. By screening title and abstracts, 44 studies were selected for full text review and 20 papers met the criteria for inclusion in this scoping review (Table 1). The final themes identified were: 1) The impact of climate change on HPM in low income countries; 2) Descriptive pieces on climate change, climate disasters, and HPM; 3) Morbidity and mortality after climate disasters in the seriously ill population; 4) Discussion of euthanasia during climate disasters; 5) Recommendations and frameworks for disaster response in the field of HPM; 6) Carbon footprint of hospices.

**Table 1:** Summary of selected articles.

Title	Author	Location	Year	Paper Type	Theme	Purpose
Lessons learned from the floods of hurricane Floyd, a disaster of biblical proportions	Lewis, B.	United States	2001	Editorial	Descriptive	Describes challenges faced by a hospice caring for patients during a hurricane
Caregiving in U.S. Gulf States During Natural Disasters and COVID-19	Boucher, N. et al	United States	2022	Qualitative	Descriptive	Describes the experiences and needs of unpaid caregivers during natural disasters including COVID 19
Mortality among Nursing Home Residents Enrolled in Hospice and Hospice Utilization Post-Hurricane Irma	Dobbs, D. et al	United States	2022	Case-Control	Morbidity and mortality after climate disasters in the seriously ill population	Calculates mortality of nursing home residents on hospice during and after Hurricane Irma in 2017 compared to same time of year in 2015.
The carbon footprint of a hospice	Dokal, K. et al	United Kingdom	2020	Mixed Methods	Carbon Footprint	Calculates the carbon footprint of an inpatient hospice unit in England
The intersection of food insecurity and health for rural Malawian women at the end of life	Dressel, A. et al	Malawi	2020	Qualitative	Impact of climate change on HPM in low resource countries	Describes the experience of women receiving palliative care in rural Malawi including how climate change impacts their quality of life
Hurricane Katrina and the legal and bioethical implications of involuntary euthanasia as a component of disaster management in extreme emergency situations	Shea, F.	United States	2010	Editorial	Discussion of Euthanasia during Climate Disasters	Analyzes the legal and bioethical considerations around involuntary euthansia during natural disasters
The changing dynamics of community care and support in rural Malawi: The impact on Women's health and wellbeing at end of life	Hawkins, M. et al	Malawi	2022	Qualitative	Impact of climate change on HPM in low resource countries	Describes the experience of Malawiam women who receiving palliative care and their caregivers, particularly how climate change affects their quality of life
Crisis Standards of Care: A Systems Framework for Catastrophic Disaster Response	Hefling, D. et al	United States	2012	Textbook	Disaster Response Recommendations	Provides recommendations and frameworks for how palliative care should be integrated into medical care during disaster responses
Surgical palliative care in Haiti	Huffman, J.	Haiti	2011	Editorial	Descriptive	Describes disaster response to the 2010 earthquake in Haiti
Long-term effects of Hurricane Andrew	Jones, J. et al	United States	1993	Editorial	Descriptive	Describes the immediate and long-term effects of Hurricane Andrew on the health and economic outcomes of the seriously ill patients
End-of-life care in natural disasters including epidemics and pandemics: a systematic review	Kelly, M. et al	Global	2023	Systematic review	Descriptive	A systematic review of end of life care in natural disasters that included articles on COVID-19 and ebola outbreaks
"I can't make all this work." End of life care provision in natural disasters: a qualitative study	Kelly, M. et al	Australia	2023	Qualitative	Descriptive	Examimes the perspectives of providers during natural disasters
Downwind from the great Tohoku earthquake: A call to global action	Koenig, K. et al	Japan	2012	Editorial	Disaster Response Recommendations	Offers recommendations to improve disaster response
Nurturing Spiritual Resilience to Promote Post-disaster Community Recovery: The 2016 Alberta Wildfire in Canada	Lalani, N. et al	Canada	2021	Editorial	Descriptive	Describes factors contributing to the recovery of a community after the 2016 Alberta wildfire in Canada
Hurricane-no power-no water-a tree on the roof! A day in the life of one creative hospice aide	Brown, L.	United States	2012	Editorial	Descriptive	Describes a single patient encounter by a hospice nurse during Hurricane Irene in 2011.
Ethical and clinical dilemmas in patients with head and neck tumors visiting a field hospital in the Philippines	Marom, T. et al	Philippines	2014	Retrospective Review	Descriptive	Describes clinical and ethical challenges for patients with head and neck tumors in the Philippines after a tropical cyclone
Emergency management: expanding the disaster plan	Ross, K. et al	United States	2007	Editorial	Disaster Response Recommendations	Makes recommendations about how healthcare organizations can create more comprehensive disaster plans
Report of the Lancet Commission on the Value of Death: bringing death back into life	Sallnow et al	United Kingdom	2022	Descriptive	Descriptive	Offers thoughts on how the climate crisis would change healthcare expenditures, particularly through incentivizing low emmision practices over high emmisision practices
Dr. Pou and the hurricane–implications for patient care during disasters	Susan Okie, M.D.	United States	2008	Descriptive	Discussion of Euthanasia during Climate Disasters	Discusses Hurricane Katrina and the accusations that seriously ill patients who were not going to be evacuated were euthanized.
Murder or mercy? Hurricane Katrina and the need for disaster training	Tyler J. Curiel, M.D., M.P.H.	United States	2006	Descriptive	Discussion of Euthanasia during Climate Disasters	Recommends changes to disaster management plans to avoid future situations where involuntary euthanasia might be considered during natural disasters

### 3.1 The impact of climate change on HPM in low-income countries

Two articles described the impact of climate change on the experience of patients nearing end of life in low-income countries. Both studies focused on women with serious illness from Malawi. The first paper interviewed 26 Malawian women who were near end of life and receiving palliative care, as well as 14 of their caregivers. [8] Interviews revealed that food insecurity was a key barrier to good quality of life and that climate change led to worsening food insecurity. In the second study, Malawian women near end of life and their caregivers were similarly interviewed, this time with a focus on community care and support [9]. Because of the low nurse to patient ratio in Malawi, women with serious illness rely on informal family or community networks as their main source of support. This study found that climate change worsened poverty through increased flooding, drought, low crop yield and community displacement. This worsening poverty reduced social cohesiveness and social support for seriously ill Malawian women.

### 3.2 Descriptive pieces on climate change, climate disasters, and HPM

Ten papers included in the review focused on describing a specific climate event. Some focused on describing an individual experience while others were qualitative analyses of semi-structured interviews and focus groups.

Jones et al. [10], Brown [11], and Lewis [12] each individually recounted specific experiences of hospice workers during natural disasters. They describe the importance of disaster planning and outlined the initial response steps, including establishing contact with hospice employees [10] and relocating patients facing housing instability or residing in unsafe facilities [11]. Locating their patients posed a substantial challenge [12], with approximately 20% of one author's patient population being unreachable, while others were displaced or physically isolated [12]. Some hospice staff were unable to commute to work, further limiting the capacity of the agencies to care for their patients [10].

Common themes across multiple articles included challenges physically accessing patients due to road damage from trees, flooding, or downed electrical wires [11,12]. Communication issues within the team arose due to a lack of phone connectivity [12]. The authors described failures of basic services such as clean water and electricity – necessary for home oxygen machines, electric beds, Hoyer lifts and other medical devices [11]. They highlighted that during climate disasters, most aid is distributed at centralized centers, posing difficulties for seriously ill patients who may be unable to wait in lines or reach these centers. [12] They shared that hospice referrals in the immediate phase of a disaster increased significantly, both because hospitals tried to maximize their acute care space and because other hospice agencies were unprepared to take new patients [10]. The articles also noted that caring for the team was essential: “each of us was experiencing a crisis. Not only did our patients and families need support, but each of us did as well” [10]. In one instance, for example, a hospice sought virtual counseling from another hospice located outside the disaster zone [10].

Two articles used semi-structured interviews or focus groups to better understand the experience of patients or care partners [13,14]. Boucher et al. found that caregivers faced multiple challenges including disruptions in their daily routines, loss of institutional supports like community-based adult daycare, complexities in disease management such as difficulty accessing care or refilling medications, and emotional and financial hardships [13]. Lalani et al. found evidence of individual and collective trauma, as well as impaired mental health. They found that spirituality, broadly defined to include connectedness with others in the community, was protective and healing [14]. Additionally, hope and faith, a sense of gratitude, compassion and altruism, and a sense of belonging were all associated with resilience [14]. Palliative care, with its focus on coping and its comfort with the connections between spirituality and medical science, holds the potential to assist a community with their recovery from a climate disaster. Palliative care uniquely recognizes the profound physical and existential suffering that emerges from clinical situations deemed incurable and unresolvable. Rather than merely acknowledging this suffering, palliative care teams actively engage with patients, instilling hope and a sense of agency. Their concerted efforts focus on what remains achievable: excellent symptom management and comprehensive, whole-person supportive care. This approach thrives on collaboration, drawing upon the diverse talents of both clinicians and nonclinician team members. Social workers, spiritual care providers, and other team members contribute to a holistic care framework that addresses physical and emotional suffering, redefines hope, and fosters spiritual resilience. Importantly, this skillset extends beyond individual patients—it holds immense value for communities grappling with the aftermath of natural disasters, where suffering is widespread and quality of life hangs in the balance. In fact, the multidisciplinary prowess of palliative care teams serves as a beacon of compassion and practical support, even within the constraints of serious illnesses and challenging climate disasters.

One article used semi-structured interviews to better understand the experience of healthcare workers providing end-of-life care during natural disasters such as wildfires, floods and the COVID-19 pandemic [15]. Kelly et al. found that healthcare workers were overextended and overwhelmed, experiencing increased workloads and dramatic role changes for which they were unprepared. Because informal support networks were disrupted, they had to take on more responsibilities for their patients. They struggled to cope with their own disaster-related fears while supporting their patients. Caregivers noticed a loss of the “human element” of care – specifically a lack of human connection due to families’ isolation from their loved ones. Finally, healthcare workers experienced a sense of powerlessness and moral distress about the care they were providing [15].

A systematic review by Kelly et al. assessed end of life care in natural disasters including epidemics and pandemics [16]. Similar themes emerged including challenges providing services, and shortages of both workforce and material resources. Consistent patterns were observed in the experiences of providers, including being forced to work in unfamiliar roles, grappling with personal fears and traumas related to the disaster, and experiencing distress over limited resources. Their review also noted challenges in obtaining opioids, a crucial medication in palliative care [16], a challenge that has been linked consistently to several post-disaster factors including inaccessible transportation routes due to impassable roads and deteriorated infrastructure, as well as a shortage of pharmacy personnel, all of which can be directly attributed to the impact of the disaster [10,11].

Other papers focused on the importance of hope [17], the ethical concerns regarding disaster response [18], and the importance of viewing end-of-life care through the lens of climate change and carbon emissions [19]. Huffman [17] acknowledged the critical significance of effective triage interventions and timely, appropriate medical treatments. However, he also underscored that there exist scenarios where these interventions alone may fall short or prove suboptimal. In such instances, Huffman advocates for a compassionate approach, urging healthcare providers to prioritize comfort and hope restoration as essential components of patient care [17]. Marom, et al. acknowledged the clinical and ethical challenges of providing the best, evidence-based care in post-disaster situations when access to said care or follow-up after care is either limited or unavailable. They highlighted the importance of cross-collaboration between the post-disaster medical team and community health resources in ensuring timely, sound medical care to patients in a post-disaster situation [18].

### 3.3 Morbidity and mortality after climate disasters in the seriously ill population

Two articles described morbidity and mortality after climate disasters. The first was an observational study that compared patients residing in nursing homes in Florida during Hurricane Irma in 2017 with the same population in 2015 at the same time of year [20]. The study found that hurricane exposure was associated with increased mortality in the 30 days immediately following the storm, both for nursing home residents enrolled in hospice and for the general nursing home population. The 90-day mortality rate was unchanged for the hospice population. For patients chronically in a nursing home, there were increased rates of hospice enrollment at 30 and 90 days after the storm [20]. In a separate article, another hospice observed a threefold increase in mortality among their population in the months following a severe hurricane [12].

### 3.4 Discussion of euthanasia during climate disasters

Three articles specifically pertaining to the concept of euthanasia during climate disasters were included in the review. All three articles referenced Hurricane Katrina, which caused severe flooding in New Orleans, Louisiana in 2005. During the flooding, hospitals had to operate under emergency conditions, facing challenges such as power outages, plumbing failures, scarce food and drinking water, insufficient human and medical resources, disrupted communication, and threats to physical safety [21]. After recovering from the disaster, one physician was accused of administering morphine and midazolam to four seriously ill patients with the intent of causing death as she believed they would not be evacuated and would consequently be left to die [22]. While she was not found guilty, her case prompted a series of perspective pieces on the role of euthanasia during disasters. This review included these articles because they addressed the ethical dilemmas arising from climate disasters, such as the possibility of euthanasia, that hospice and palliative medicine providers may be called upon to address.

The term euthanasia has fallen out of favor in the medical literature [24]. Instead, the term Medical Aid in Dying (MAiD) is used to describe the process where patients with a terminal illness request medications that will lead to their death, typically out of a need for symptom relief or for a sense of control [25]. We choose to use the term euthanasia instead of the term MAiD here because MAiD implies a process initiated and controlled by the patient. In the situation of a climate disaster where there are scarce resources, this process is unlikely to be initiated by the patient and the term MAiD would be inaccurate.

Fredericka Shea provided a bioethical and legal analysis, including a US-based legal history of major rulings that have influenced how we perceive withdrawal of life-sustaining treatments [23]. She identified the ethically permissible concept of double effect, which is the intent to relieve symptoms where death or an increased risk of death is an understood but undesired consequence. This is contrasted with her definition of euthanasia as the intentional act of killing a patient as a method to relieve suffering [23]. Her perspective piece argued strongly against euthanasia in disaster settings: “Involuntary euthanasia is directly contrary to the established principles of patient autonomy and self-determination and inevitably requires a determination by the physician of what is an acceptable quality of life for the patient” [23]. Okie writes similarly about intent being key: ”If her intent was to relieve suffering…then I don't think anybody in the ethics community would bat an eye. If it [was] specifically to hasten death . . . then it becomes a little more questionable” [22]. Okie also called for training on triage skills, like what is provided in the US uniformed health services training [22]. Curiel voiced similar perspectives, particularly on the importance for a group decision-making process and disaster preparedness training [21].

### 3.5 Recommendations and frameworks for disaster response in the field of HPM

Three articles focused on disaster response as it related to the field of HPM. The National Academies of Science and the Institute of Medicine published a framework for crisis standards of care [26] which drew on insights from previous disasters and aimed to guide agencies and healthcare leaders in creating standards of care for disaster scenarios. They devoted a section of their publication to palliative care, stating that “the provision of palliative care in the context of a disaster with scarce resources can be considered a moral imperative of a humane society" [26]. They set an expectation that all patients receive care in disaster situations, regardless of resource availability, and acknowledged that this may include palliative or end-of-life care. They recommended integration of multi-disciplinary palliative care teams across all levels and settings of healthcare delivery, including the adoption of evidence-based palliative care guidelines to crisis standards of care. They note that the public would benefit from a better understanding of palliative care to prevent a sense that it involves abandoning patients or deliberately causing death. Finally, they recommend involving palliative care in the development of triage models and increasing palliative care surge capacity through education and training [26].

On a more granular level, Ross et al. discussed the development of comprehensive emergency management plans [27]. They recommended groups undergo a hazard vulnerability analysis (HVA) which they defined as an assessment of all potential risks to patient care and service delivery that might arise in a disaster. They recommended developing action plans for each risk, separated into preparation, response and recovery phases. Examples of preparation type activities include designating roles and responsibilities for all staff in the disaster setting, establishing a clear chain of command, and developing a triage process so that providers can find the sickest patients easily. Examples of response activities included providing box fans to patients during extreme heat emergencies and evacuating staff or patients from unsafe facilities. For the recovery phase, example behaviors might include locating displaced staff and patients, replacing lost inventory, or restarting routine services [27].

Koenig et al. shared their experience after an earthquake in Japan and echo the previous articles’ calls for a multi-disciplinary approach to disaster response [28].

### 3.6 Carbon footprint of hospices

A study conducted by Dokal investigated the carbon footprint of a 16-bed, inpatient hospice in southwest England, including emissions from staff, patient and family commutes [29]. The findings revealed an annual carbon footprint of 420 tons of carbon dioxide (CO2), most of which was from travel (35%), gas (33%), and non-medical supplies (17%). To provide context, they compared this figure to the average carbon footprint of a person in the UK (10 tons CO2) and the carbon footprint generated by a single cataract surgery (405 tons CO2). They also surveyed the hospice staff and found high support and enthusiasm for environmental sustainability [29]. Notably, there is a lack of studies assessing the carbon footprint of hospice care provided at a patient's home, which is the more common approach in the United States.

## 4 Narrative review

To supplement our review, we reached out to colleagues through formal and informal networks to gather perspectives from HPM providers who have firsthand experience with care affected by climate change. We specifically included two narratives to provide a range of perspectives. The first narrative describes the impact of wildfires on hospice care, providing a counterbalance to existing literature that predominantly emphasizes flooding and meteorological climate events. The second narrative examines the repercussions of flooding on palliative care in Africa, offering a more comprehensive and unique perspective that addresses a gap in the literature, which tends to focus on English-speaking and high-income countries.

### 4.1 Impact of wildfires on hospice and palliative care [31]

The Camp Fire sparked on November 8th, 2018, roaring through the small foothill communities of the Sierra Nevada Mountains, destroying the town of Paradise, California. At the time, it was the deadliest, most destructive fire in California history. Eighty-six people lost their lives in the fire; many more subsequently died. However, anecdotal reports from colleagues indicate that patients who were under the care of hospice or home health were successfully evacuated.

The palliative care program in the neighboring hospital saw several survivors evacuated from the fire. They had fled in such a hurry that medications and lifesaving equipment were abandoned. There were patients in acute respiratory failure when they ran out of oxygen support. There were also patients in cardiac distress and others experiencing injuries and interrupted cancer care. Patients had acute strokes as their coumadin management was upended. Several of the patients did not survive. Attempts to report these deaths as fire-related went unresolved; there was no formal mechanism to count these patients.

The medical community in the impacted areas ceased functioning as personnel fled for safety. A hospital that was evacuated that morning has never re-opened. Access to primary and specialty care services remained challenging for years to come. The community also lost access to the only inpatient hospice facility in the area. Fire survivors camped in a vacant lot near the Walmart on the edge of town. Volunteers from the neighboring hospital went to the camps to assist those who needed medical evaluations and prescription refills.

For many of the survivors, the disaster is ongoing, more than five years later. Many patients who were evacuated or lost homes experienced exacerbations of their medical issues. This was especially notable for those with dementia. Providers noted rapid progression and earlier demise among their patients, especially for those who were relocated multiple times. They also saw clinical declines in patients with heart disease and pulmonary issues which persisted due to an overall decline in the air quality. The abuse of alcohol, opioids and other substances remain major challenges in the county. The smell of smoke and blustery winds trigger survivors who are dealing with PTSD, anxiety and depression.

The local hospices continue to see the impacts of the fire. Many of the survivors remain in unstable environments, living in RVs and trailers, still awaiting financial settlements. The homes that have been rebuilt lack the usual personal artifacts of a lifetime, such as family pictures on the wall. These homes have a sterile feel. When it comes to meaning-making at the end of life, these families lack the usual mementos that might trigger memories or would be passed along to the next generation. As they approach the end of their lives, patients often need to retell their memories of the fire. It is a collective trauma shared by the community and speaks to the need to incorporate trauma-informed care approaches when working with those impacted by climate disasters. The work of palliative care is often described as “walking into the worst day of someone's life”; this was especially true during the Camp Fire.

### 4.2 Impact of flooding on hospice and palliative care in Nigeria [32]

Flooding, storms, droughts, heat waves, and other rapidly developing environmental traumas are some of the most common climate events occurring in low and middle-income countries such as Nigeria. People with serious illnesses, especially those receiving end-of-life care, are among those most vulnerable during disasters and extreme weather conditions. In Nigeria, there has been an increase in the burden of chronic diseases requiring palliative care, such as cancer and HIV/AIDS, due to longer life expectancies and urbanization. Providing palliative care is challenging due to underdeveloped road networks, communication gaps, and disrupted energy and water infrastructure, all of which are exacerbated simultaneously by climate change-related disasters. Such infrastructure may already be compromised as a result of years of neglect, a lack of funding for basic upkeep and improvements, corruption, understaffing, or major world events like war and political instability. These challenges significantly affect the quality of life for seriously ill patients and their household members through limited access to medication and food, home damage, displacement, and reduced access to medical services and treatments. They also affect organizations that offer palliative care services as well as the employees and volunteers of those organizations. They lower clinical efficiency to a major degree and sometimes completely halt the delivery of services. This situation adds further pressure to the healthcare profession which is already understaffed in the areas of palliative care and hospice medicine.

## 5 Discussion

The intersection of climate change and HPM remains an under-researched topic, with most of the available literature being descriptive and focused on specific climate disasters. In both the existing literature and the narratives provided by members of the HPM community, common challenges to providing care were identified. Widespread issues included difficulties physically reaching patients and logistical challenges accessing medication or medical equipment [10,13,16]. Patients experienced isolation and loss of social or community support [14]. Unsurprisingly, both hospice referrals and mortality increased during climate disasters and in the period immediately following them [12,20].

Providers experienced moral distress from a complex and intersecting set of challenges. Providers were often asked to take on new roles and responsibilities for which they felt unprepared [15]. At the same time, they were also personally impacted by the climate-induced disaster, limiting their ability to focus on patient care [10]. This, combined with the challenges that patients experienced, often led to reduced quality of care, which caused significant distress for providers [15].

Preparedness was seen as a key component in providing better care during climate events. While the timing of climate events remains unpredictable, they are certain to occur and therefore lack of preparedness at either the organizational or national level is unjustifiable. Organizations can prepare by creating comprehensive disaster management plans [27], by training providers in how to respond during disasters [22], and by creating sets of policies to provide guidance [23]. At the national level, enhanced training in palliative care, the establishment of structures facilitating coordination among local organizations, and increased public education regarding the role of palliative care have been identified as crucial steps [26]. Lastly, quality data on morbidity and mortality during climate events is essential to understand the full impact of climate change on our patients and the effectiveness of our response. To achieve this, systems must be created to gather these data.

Part of disaster response involves the triaging of scarce resources. One article mentions that Do Not Resuscitate (DNR) orders were used to inform triage decisions [22]. We would emphasize the importance of a nuanced understanding of what DNR means. While this can vary in different states or countries, there is typically a distinction between DNR and comfort measures only (CMO) or comfort care. DNR typically indicates that a patient has shared specific wishes about the care they would like to receive if their condition worsens to the point of cardiac arrest – specifically that they would not want providers to attempt CPR and resuscitation in that setting. It typically does not specify the patient's wishes for any condition less severe than cardiac arrest, including ventilatory support, use of vasopressors, hemodialysis, or other forms of life support. In contrast, CMO indicates a patient's wish to focus purely on measures to keep them comfortable, avoiding interventions that prolong their life if these interventions were to cause discomfort. Patients often fear that providers will read DNR as “Do not treat” [30]. In disaster situations, triaging by DNR status would be against patients’ wishes. In contrast, incorporating the existence of CMO into triage decisions could be seen as honoring patient's wishes.

Although euthanasia does not need to be a part of a triage process, debate over its appropriateness appeared in our literature search. While euthanasia is not a routine part of hospice and palliative medicine practice, articles related to the topic were included in our review because it is possible that hospice and palliative medicine providers could be consulted in decisions about euthanasia. All three articles included in the review opposed any form of euthanasia in the context of climate disasters, a position strongly supported by the authors of this review. Palliative care has a role in providing excellent symptom management in disaster settings. While the medications used to provide comfort are the same as those used to hasten death, our collective experience is that these medications can provide comfort without hastening death when dosed by standard guidelines. The unspoken argument in favor of performing euthanasia is rooted in the assumption that for patients inevitably facing death, hastening their demise with medications will prevent suffering. Our counterargument is that with effective palliative care, suffering can be prevented with minimal to no impact on expediting death. Therefore, we advocate against euthanasia and for excellent palliative care for all patients in disaster scenarios.

Our study had several limitations. There were few studies related to our research objective. We limited our review to literature published in English, potentially overlooking significant literature on how climate change impacts non-English speaking regions. The majority of articles in our review came from high-income countries but climate change has a disproportionately greater impact on low-income nations. We chose to exclude studies on epidemics or pandemics including COVID-19 data, and focused instead on weather-related climate disasters, which distinguishes this review from previous ones [16]. Because the majority of the selected articles are qualitative in nature, they possess the strengths and limitations inherent to qualitative data. While they provide rich, detailed data on the lived experience of individuals, they are susceptible to bias and would be complemented by robust data using rigorous quantitative methodologies. Additionally, because each climate-induced disaster involves unique circumstances, it is unclear how information from firsthand accounts is generalizable. The majority of articles identified in our review are descriptive in nature, often presenting firsthand accounts of healthcare workers’ experiences. Most data still focus on response to climate-related disasters such as hurricanes, floods, or wildfires. While this is valuable given that climate-induced disasters are projected to become increasingly common, it also imposes limitations. Climate change undoubtedly affects the delivery of hospice and palliative care outside of climate-driven events, but our scoping review did not identify literature that addresses this.

This scoping review reveals a number of areas ripe for future research. Research on the impact of climate change on palliative care remains mostly anecdotal and descriptive. Future research involving more quantitative methodology would help clarify the broader experience in the field. Moving forward, intervention-based research will be necessary to improve the care provided to seriously ill patients during climate events. Palliative care focuses on whole-person care and includes the social and emotional health of those within a patient's support system. As such, caregiver outcomes such as caregiver mental health, PTSD, and complex grief are relevant to the field of palliative care [33]. Climate change can affect the grief process through loss of community and loss of communal rituals that support bereavement. As Lalani et al. discussed, reconnection with the community at large is an important factor in resilience [14]. and more research on the impact of climate change on bereavement will be essential to provide evidence based interventions on the community level. We also acknowledge that the focus on climate-related events is relatively narrow in scope and that research on the impact of climate change outside this setting is essential to better understand the total impact of climate change on the field of hospice and palliative medicine. Additionally, no article specifically addressed the impact of climate change on children with serious illnesses, or on the provision of pediatric palliative care. While we expect that many of the challenges and barriers to access that occur with climate change will affect pediatric populations as much as they will affect adult populations, we also hypothesize that there will be unique challenges that arise in pediatric populations dealing with climate change, and that research focusing on this topic will be essential to characterize these challenges***.***

## 6 Conclusion

There has been little research on the impact of climate change on the field of hospice and palliative medicine, with most of the existing literature being descriptive in nature and focused on climate-related disasters such as hurricanes, floods, or fires. During such events, seriously ill patients, their care givers, and the medical teams face significant challenges. In response to this crisis, the field of hospice and palliative care has a critical role to play. The skills required to deliver bad news and stay present, to show patients how to cope with the unacceptable, and to holistically approach suffering all can be applied to the complexities and distress of climate change. The palliative community can thus embody the ethos of compassionate care in the face of an ever-evolving global crisis to advocate for just transitions and equitable solutions.

## CRediT authorship contribution statement

**David Harris:** Writing – review & editing, Writing – original draft, Project administration, Methodology, Formal analysis, Data curation, Conceptualization. **Bhargavi Chekuri:** Writing – review & editing, Writing – original draft, Formal analysis. **Aldebra Schroll:** Writing – review & editing, Writing – original draft, Project administration, Investigation, Formal analysis, Data curation, Conceptualization. **Nisha Shah:** Writing – review & editing, Writing – original draft, Methodology, Data curation, Conceptualization. **Laadi Swende:** Writing – review & editing, Writing – original draft, Methodology, Formal analysis, Data curation, Conceptualization. **Collins Uzuegbu:** Writing – review & editing, Writing – original draft, Methodology, Formal analysis, Data curation, Conceptualization. **Pamela Young:** Writing – review & editing, Writing – original draft, Methodology, Formal analysis, Data curation, Conceptualization.

## Declaration of competing interest

The authors declare that they have no known competing financial interests or personal relationships that could have appeared to influence the work reported in this paper.
